# Etude des connaissances, attitudes et pratiques en matière de réintégration sociale des femmes victimes de fistule obstétricale: région de l'Extrême-nord, Cameroun

**DOI:** 10.11604/pamj.2015.20.172.5959

**Published:** 2015-02-24

**Authors:** Sanou Sobze Martin, Sali Ben Béchir Adogaye, Mabvouna Biguioh Rodrigue, Douryang Maurice, Teikeu Tessa Vladimir Vivaldi, Saah Fopa Michael Amede, Ovaga Eyenga Landry Marie, Ausseil Sandra Meriam, Vittorio Colizzi, Russo Gianluca

**Affiliations:** 1 Département des Sciences Biomédicales, Faculté des Sciences, Université de Dschang, Cameroun; 2 Faculté de Médecine, Pharmacie et d'odontostomatologie, Université Gamal Abdel Nasser, Conakry, Guinée; 3 Faculté des Sciences de la Santé, Université Abdou Moumouni, Niamey, Niger; 4 Association Eit Tiddeye, Niamey, Niger; 5 Université de Rome “Tor Vergata”, Rome, Italie; 6 Département de Santé Publique et de Maladies infectieuses, Faculté de Médecine et Pharmacie “Sapienza”, Université de Rome, Italie

**Keywords:** Fistule obstétricale, réintégration sociale, Extrême-nord, Cameroun, obstetric fistula, Social integration, far-north, Cameroon

## Abstract

**Introduction:**

La fistule obstétricale est un orifice entre le vagin et la vessie ou le rectum, voire les deux. Ses impacts sont des conséquences anatomo-fonctionnelles et sociales. On estime à plus de 19 000 le nombre de femmes qui souffrent de fistule obstétricale au Cameroun.

**Méthodes:**

Il s'agissait d'une étude transversale descriptive conduite dans trois districts de santé de la région de l'Extrême-nord. Vingt-huit femmes victimes de fistules obstétricales, quarante-deux membres de leur entourage et vingt-quatre agents de santé ont été interviewés entre Novembre et Décembre 2013. Trois types de questionnaires ont été utilisés. Les données ont été analysées dans Epi Info version 7.1.4.0. Les moyennes et les fréquences ont été calculées avec un intervalle de confiance à 95%.

**Résultats:**

46,4% des femmes victimes de fistule obstétricales interviewées avaient subi une intervention chirurgicale réparatrice parmi lesquelles, 61,5% bénéficiaient de la réintégration. Le fonds de commerce (62,5%) était l'aide la plus reçue. Vingt-deux membres de l'entourage savaient pourquoi on fait la réintégration. Selon eux, les considérations socioculturelles (68,2%), sont la principale barrière de la réintégration. D'après les agents de santé, le suivi psychosocial (58,3%) est la principale activité de la réintégration dans les centres de prise en charge de la fistule.

**Conclusion:**

La prise en charge des fistules obstétricales au Cameroun souffre de manque de réintégration sociale. Ceci expliquerait en partie la persistance de cette pathologie. Un accent devrait être mis sur l'appui matériel, financier et sur le suivi psychosocial des femmes victimes de fistule obstétricale.

## Introduction

La fistule obstétricale (FO) est une lésion du tissu pelvien causée par un accouchement prolongé et difficile enl'absence de soins obstétricaux appropriés pour y remédier [1–3]. Littéralement il s'agit d'un orifice entre le vagin et la vessie ou le rectum, voire les deux, qui provoque une incontinence urinaire et/ou fécale chronique ayant des effets nocifs sur la vie sociale et l’état de santé de la femme [4–6]. Selon l'Organisation Mondiale de la Santé (OMS), environ 800 femmes meurent chaque jour dans le monde du fait de complications liées à la grossesse ou à l'accouchement [7]. En 2013, 289 000 femmes sont décédées pendant ou après la grossesse ou l'accouchement [7]. La quasi-totalité de ces décès (99%) se sont produit dans des pays en développement, dont plus de la moitié en Afrique subsaharienne (57%), ce qui fait de l'Afrique la région du monde où le ratio de mortalité maternelle est le plus élevé [7–10]. Pour chaque femme qui meurt, 20 à 30 femmes souffrent d'une maladie ou d'une invalidité à court ou à long terme, notamment une grave anémie, un dommage causé aux organes génitaux, une douleur chronique, la stérilité ou une sérieuse invalidité post-partum comme la FO [11, 12]. Par ailleurs, santé maternelle et santé néonatale étant étroitement liées, près de 3 millions de nouveau-nés meurent chaque année donc 12,99% dans les pays en voie de développement [13, 14]. On dénombre également 2,6 millions d'enfants mort-nés chaque année [15]. En outre, environ 40% de tous les décès d'enfants de moins de cinq ans sont des décès néonataux [14]. On estime à environ 2 à 3,5 millions le nombre de femmes souffrant de manière permanente des problèmes liés à la FO dans les pays en développement, avec 50 000 à 100 000 nouveaux cas chaque année [16, 17]. Pratiquement éradiquée dans les pays développés, la FO continue à faire des ravages dans les pays pauvres notamment ceux de l'Asie et de l'Afrique subsaharienne [16–18]. Au Cameroun, c'est plus de 19 000 femmes qui souffrent de FO, la majeure partie se trouvant dans la région de l'Extrême-nord [19]. Les impacts liés à la FO sont d'une part des conséquences anatomo-fonctionnelles (infections à répétition, stérilité, infirmité sexuelle, incontinence urinaire et/ou fécale). D'autre part des conséquences sociales telles que l'exclusion sociale, rejet du mari, stigmatisation, discrimination et une pauvreté iatrogène suite aux dépenses catastrophiques [20–22].

Le Cameroun s'est engagé dans une campagne de chirurgie des FO à travers diverses activités soutenues par ses partenaires techniques et financiers. Ces activités comprennent entre autre la prévention, la prise en charge chirurgicale, mais très peu de réintégration sociale des femmes opérées [23]. Pourtant, une enquête menée auprès de 99 femmes dans le service de Maternité de l′Hôpital provincial de Maroua entre Mai 2005 et juillet 2005 a montré qu'une femme sur trois interrogées pense qu'il faille se cacher et une femme sur dix suggère qu'il faille se suicider en cas de FO [24]. En tant que processus, la réintégration sociale permet aux femmes opérées de bénéficier d'une formation, des activités génératrices de revenue (AGR) et d'un capital qui leurs permettra de mettre en pratique la formation reçue une fois retournées dans leurs villages d'origines. Cette mission apparemment anodine, n'incombe pas seulement aux autorités sanitaires mais aussi à toute la société civile. En effet la réintégration sociale doit impliquer les leaders communautaires ainsi que les organisations à base communautaires (OBC) qui sont appelés à apporter un appui de proximité. Toutefois, le processus doit tenir compte de l'avis et des connaissances des femmes bénéficiaires afin de mieux comprendre leurs besoins, leurs attenteset leurs conceptions de la réintégration sociale. Afin d'atteindre cet objectif, nous avons mené une étude sur les connaissances, attitudes et pratiques en matière de réintégration sociale auprès des femmes victimes de fistule obstétricale (FVFO) dans la région de l'Extrême-nord, Cameroun.

## Méthodes

Il s′agissait d′une étude transversale descriptive conduite dans trois districts de santé (Doukoula, Moulvoudaye, et Yagoua) de la région de l'Extrême-nord, Cameroun entre Novembre et Décembre 2013. Ces districts de santé ont été choisis parce que c'est delà que proviennent la majorité des FVFO. L’étude a consisté au recueil des données sur les connaissances, attitudes et pratiques sur la réintégration sociale des FVFO. L'enquête a été conduite auprès des FVFO, leur entourage et le personnel socio-sanitaire intervenant ou non dans la prise en charge de la FO. Les femmes interrogées sont issues du répertoire régional des femmes victimes de fistule obtenu à la Délégation Régionale de la Promotion de la Femme et de la Famille. Ce répertoire comptait cent (100) femmes reparties dans toute la région. Les femmes ayant refusées de participer à l’étude n'ont pas été enregistrées, ni dénombrées. Pour le recueil des données, trois types de questionnaires ont été administrés par interview direct. Ils comportaient des questions fermées et ouvertes auxquelles un code a été attribué. Les données ont été entrées dans l'ordinateur à l'aide du logiciel Microsoft Office Excel’ 2013, et importés dans Epi Info version 7.1.4.0 (CDC, Atlanta, GA, USA) pour analyse. Les femmes n'ayant pas répondu intégralement à toutes les questions ont été exclues de l'analyse. Après dépouillement, les données de vingt-huit femmes sur quarante-deux interrogées, de quarante membres de l'entourage et de vingt-quatre agents de santé ont été analysées. Les moyennes et les fréquences ont été calculées avec un intervalle de confiance à 95%.

## Résultats

**Figure 1:**
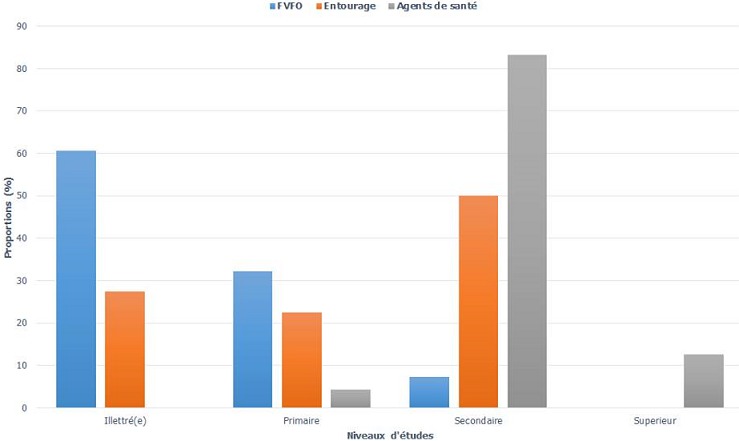
Répartition des niveaux d’étude des participants

**Description de l’échantillon:** l’âge moyen des FVFO était de 44 ans (range: 20 ‘ 84 ans), 67,9% d'entre elles étaient mariées, 25% veuves et le reste (7,1%) étaient séparées ou divorcées. Chez l'entourage et les agents de santé, les moyennes d’âge étaient de 36 ans (range: 14 ‘ 73 ans) et 35 ans (range: 22 ‘ 48 ans) respectivement. Les personnes de l'entourage des FVFO étaient des membres de la belle-famille (47,5%), les conjoints (25%), un parent (4%), un frère/s'ur (4%) ou un(e) ami(e) (3%). Concernant le niveau d’étude, la plupart des FVFO étaient illettrées (60,7%), 50% de leur entourage et 83,3% des agents de santé avaient un niveau d’étude secondaire respectivement (Figure 1). L’étude de l'occupation professionnelle a montré que 39,3% des FVFO étaient des commerçantes, 32,1% sans emploi et 28,6% étaient cultivatrices ou éleveuses. Pour ce qui est de leur entourage, 67,5% étaient sans emploi, 12,5% commerçants, 7,5% employés du secteur public contre 5% du secteur privé et 7,5% étaient cultivateurs ou éleveurs. Parmi les agents de santé, on dénombre un médecin, dix aides-soignants, dix infirmiers et trois agents santé communautaires.

**Figure 2:**
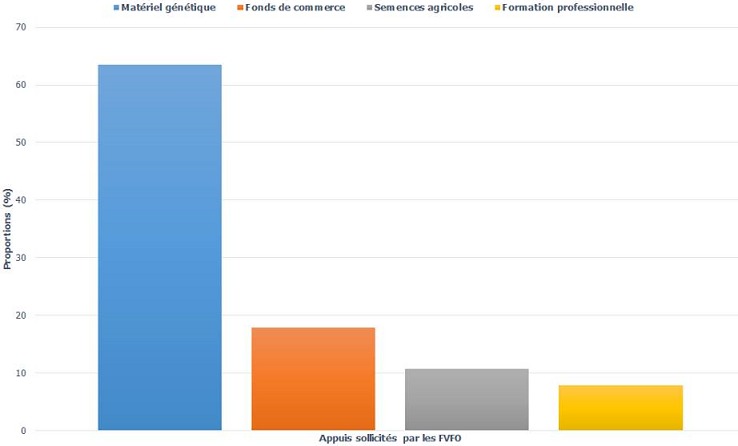
Principales appuis sollicités par les femmes victimes de fistule obstétricale

**Connaissances des FVFO en matière de réintégration sociale:** parmi les 28 FVFO interviewées, 46,4% avaient déjà subi une intervention chirurgicale de réparation principalement à l'hôpital régional de Maroua. Ces dernières avaient été informées de la réintégration sociale, en revanche 61,5% en étaient bénéficiaires. Le fonds de commerce (62,5%) et le matériel génétique d’élevage (37,5%) sont les principaux appuis reçus par celles-ci. Tous ces appuis ont été développés en AGR, et toutes les bénéficiaires en sont satisfaites. D'une manière générale, toutes les femmes enquêtées pensent que la réintégration sociale est une activité bénéfique pour la FVFO, 64,3% savaient pourquoi on fait la réintégration sociale et 42,9% connaissaient une femme qui en avait déjà bénéficié. Le matériel génétique pour l’élevage de petits ruminants est l'aide la plus sollicitée (63,5%), suivi par le fonds de commerce (17,9%), les semences agricoles (10,7%) et en fin par la formation professionnelle (7,9%) (Figure 2). Toutes les femmes déclarent que ces aides permettrons à gagner leurs vies, toutefois 28% d'entre elles ont affirmé que cela ne leur fera pas oublier leur situation de porteuse de FO. La plupart des femmes (75%) pensaient que leurs maris seront favorables à leur inclusion dans le processus de réintégration sociale, 7,2% envisageaient plutôt un refus de leur part et 17,8% étaient indécises quant à la réaction de leurs maris. De même, 50% des femmes pensaient que leurs communautés d'origine accepteront les aides, 28,6% étaient indécises, 10% pensaient au rejet, également 10% pensaient à une indifférence de leurs communautés vis-à-vis des appuis reçus.

**Connaissances de l'entourage des FVFO en matière de réintégration sociale:**vingt-cinq personnes de l'entourage des FVFO étaient informées de la réintégration sociale, parmi lesquelles 22 savaient pourquoi on fait la réintégration sociale et tous ont affirmé qu'il s'agissait d'une activité bénéfique pour les FVFO. Pour ces derniers, la réintégration sociale est une activité qui vise principalement à aider la femme à: mieux se sentir dans son foyer (68,2%), accepter son statut de porteuse de fistule (18,2%), subvenir à ses besoins (9,1%), enfin, à se faire accepter dans la communauté (4,1%) (Tableau 1). Toutefois dans l'optique d'atteindre un résultat optimal, ils suggèrent d'impliquer les conjoints des femmes dans le processus de réintégration (50%), d'orienter les aides en fonction des besoins des femmes (36,4%) et de prendre la femme en charge avec toute sa famille (13,6%). Aussi selon eux, les considérations socioculturelles (68,2%), l'absence de suivi des femmes (18,2%) et le non implication des autorités traditionnelles et religieuses (13,6%) représentent les principales barrières auxquelles la réintégration pourrait se heurter (Tableau 2). Enfin, l'ensemble des personnes de l'entourage de FVFO préfèrent le commerce (92,5%) et l’élevage de petits ruminants (7,5%) comme activités de réintégration sociale.

**Tableau 1:** Opinions des personnes de l'entourage sur la réintégration sociale des FVFO

Opinions (N= 22)	n	%
Activité aidant la femme à accepter son statut de porteuse de fistule	4	18,2
Activité aidant la femme à se faire accepter dans la communauté	1	4,5
Activité aidant la femme à subvenir à ses besoins	2	9,1
Activité aidant la femme à mieux se sentir dans son foyer	15	68,2

N: nombre total d'observations; n: nombre d'observations par catégorie;%: pourcentage

**Tableau 2:** Principales barrières de la réintégration sociale des FVFO selon les personnes de l'entourage

Barrières (N= 22)	n	%
Non implication des autorités traditionnelles/religieuses	3	13,6
Absence de suivi des femmes	4	18,2
Culture/tradition/mœurs	15	68,2

N: nombre total d'observations; n: nombre d'observations par catégorie;%: pourcentage

**Figure 3:**
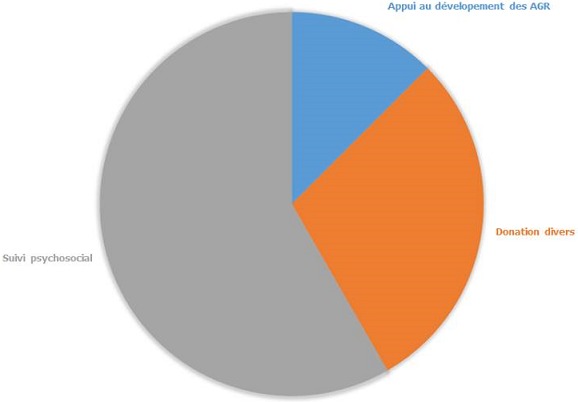
Activité de réintégration sociale mise en œuvre dans les centres de prise en charge de la fistule obstétricale selon les agents de santé

**Connaissances des agents de santé en matière de réintégration sociale:** la moitié des agents de santé (50%) interviewés au cours de cette étude participaient directement à la réintégration sociale des FVFO. Les avis de tous les agents de santé recueillis en ce qui concerne le processus de réintégration social des FVFO relèvent que les activités actuellement mises en ‘uvre dans les structures de prise en charge comprennent: le suivi psychosocial (58,3%), les donations diverses (29,2%) et des appuis au développement des AGR (12,5%) (Figure 3). Toujours selon eux, près de 83% de FVFO ayant bénéficié de la réintégration sociale sont acceptées une fois retournées dans leurs villages d'origine. Les limites actuelles de la réintégration sociale évoquées par les agents de santé sont: la mauvaise gestion des aides au niveau des centres de prises en charge (41,7%), la faible adhérence des femmes (25%), la non implication des autorités traditionnelles et religieuses (16,7%) et les obstacles socioculturels (12,5%). Ainsi, les suggestions faites par les agents de santé pour l'amélioration de cette activité sont: l'implication de tous les acteurs sociaux (autorités traditionnelle et religieuse, organisations à base communautaire, les époux, les proches des FVFO, etc.) dans le processus de réintégration (58,3%), la prise en charge des FVFO avec toutes leurs familles (29,2%) et l'octroi des appuis au développement des AGR en fonction des besoins de chaque bénéficiaire (12,5%).

**Activités suggérées en vue d'améliorer la réintégration sociale des FVFO:** afin d'améliorer la réintégration sociale des FVFO, les avis de l'ensemble des participants de cette étude ont été recueillis demanière à formuler les recommandations les plus adaptées. D'une manière globale, on retient que le suivi sanitaire (27,2%), l'appui financier (26,1%), le suivi psychosocial (19,6%), l'octroi des AGR (13%), la création des comités locaux de gestion des aides (13%) et l'appui logistique (1,1%) sont les principales activités à intégrées dans le processus de réintégration sociale des FVFO (Tableau 3).

**Tableau 3:** Activités suggérées par les participants en vue d'améliorer la réintégration sociale

Activités suggérées	FVFO N= 28 n(%)	Entourage N= 40 n(%)	Agents de santé N= 24 n(%)	Total N= 92 n(%)
Suivi psychosocial	1(3,6)	8(20)	9(37,5)	18(19,6)
Suivi sanitaire	6(21,4)	11(27,5)	8(33,3)	25(27,2)
Appui financier	11(39,3)	12(30)	1(4,2)	24(26,1)
Octroi des AGR	2(7,1)	4(10)	6(25)	12(13)
Appui logistique	1(3,6)	0	0	1(1,1)
Comités locaux de gestion	7(25)	5(12,5)	0	12(13)

N: nombre total d'observations; n: nombre d'observations par catégorie;%: pourcentage

## Discussion

Notre étude visait à faire une analyse situationnelle des connaissances, aptitudes et pratiques en matière de réintégration sociale des FVFO dans la région l'Extrême-nord, Cameroun. Pour ce faire, nous avons enquêté les femmes opérées ou porteuses de FO, leur entourage et les agents de santé. La FO étant une affection à caractère stigmatisant et discriminatoire [25], très peu de femmes se déclarent comme en étant porteuse de peur d’être l'objet de moquerie, de rejet ou de toute sorte d'humiliation [25–30]. Par conséquent, les femmes qui ont participé à cette étude ont été retrouvées à partir du répertoire des FVFO de la Délégation Régionale de la Promotion de la Femme et de la Famille de l'Extrême-nord. Par ailleurs, très peu d’études ont été conduites sur la problématique de la réintégration sociale des FVFO, ainsi la discussion de notre thématique s'est basée sur des études portant sur la FO en général.

**Description de l’échantillon:** contrairement à d'autres études qui présentent la FO comme étant un facteur de divorce ou de séparation [31–34], la majorité (67,9%) des FVFO de cette étude étaient mariées. Cette situation assez particulièrepeut être attribuée à des nombreuses campagnes d’éducation et de communication pour le changement de comportement (CCC) en matière de FO qui sont conduites dans la région de l'Extrême-nord [35]. L'intensification des campagnes de sensibilisation pourrait donc aider à lutter efficacement, voire à éradiquer la FO. Les conjoints (25%) et les membres de la belle-famille (47,5%) des femmes ont acceptés de participer à l’étude. Ce qui montre que la question de la FO est abordée au niveau du couple et/ou familial et témoigne aussi d'un changement de comportement profond vis-à-vis de la FO, de l'acceptation de la femme comme porteuse de fistule, et d'un changement de perception de la FO qui n'est plus perçue comme une malédiction ou une pathologie d'origine mystique [35]. De nombreuses études ont déjà montré que l'analphabétisme et la pauvreté étaient des facteurs de risque de survenue de la FO [36–38]. Nous sommes arrivés à la même observation. Dans cette étude, la plupart des FVFO étaient illettrées (60,7%), sans emploi (32,1%) ou exerçaient une petite activité de subsistance (commerce, élevage). On pense en effet que, lorsqu'une femme est instruite, elle a plus de chance d′avoir une bonne culture sanitaire, ce qui larendra consciente de l′importance des soins obstétricaux de qualités et luipermettra d′adopter un meilleur comportement vis-à-vis de la médecine moderne. Quant à une activité économique rémunérée, elle constitue une source de revenu supplémentaire pour le ménage et pour la femme, nécessaire à l′achat des biens et services relatifs aux soins de santé. Ainsi,le développement socio-économique est un aspect non négligeable dans la lutte contre la FO en milieu défavorisé.

**Connaissances en matière de réintégration sociale des FVFO:** la plupart des femmes souffrant de FO sont originaires des zones rurales très reculées ayant un accès limité à l'information, ce qui peut expliquer le fait que peu de femmes (46,4%) aient bénéficié d'intervention chirurgicales qui se déroulent à l'Hôpital Régional de Maroua dans le cadre des campagnes de réparation la FO [19, 32]. La totalité des femmes opérées ont été informées de la réintégration sociale, cependant 38,5% d'entre elles n'en bénéficiaient pas. En effet, la plupart des interventions de prise en charge de la FO sont des opérations chirurgicales de réparation et à base hospitalière. Pourtant, les FVFO ont besoin d'une assistance post opératoire particulière afin de surmonter le traumatisme psychologique d'avoir été rejetées par leurs familles et communautés et de démarrer une AGR [30]. Dans les cas particuliers de non guérison, où la fistule est très complexe pour une réparation définitive et où l'incontinence (urinaire/fécale) est permanente, la patiente aura besoin non seulement d'un suivi médical permanent, mais aussi d'un suivi psychosocial et d'une aide financière. Dans cette étude 64,3% des femmes interviewées savaient pourquoi on faisait la réintégration sociale, mais seulement 42,9% de celles-ci connaissaient une femme réintégrée. Or, les femmes opérées ayant été réintégrées doivent être utilisées comme des éducatrices afin d'encourager d'autres femmes porteuses de fistule à adhérées aux programmes de prise en charge mis en œuvre dans leurs communautés. C'est l'une des approches innovatrices utilisées par la Foundation for Women's Health Research and Development (FORWARD), qui consiste à intégrer les femmes réintégrées dans la mise en œuvre des activités du projet au niveau de leurs villages d'origine. Ce qui améliore le suivi de l’évolution des AGR au niveau communautaire [39]. Malgré ce manquement, 72% des FVFO de cette étude pensent que la réintégration sociale est une activité bénéfique pour elles. Comme lesFVFO interviewées, plus de 60% de leur entourage avait été informé de la réintégration sociale et 88% de ces derniers pensaient que cette activité est bénéfique pour la femme porteuse de fistule. Le fonds de commerce était l'aide la plus sollicitée par ceux-ci. Cependant à cause de l'insuffisance des ressources en partie liée aux problèmes de mauvaise gestion, la réintégration sociale des femmes se fait majoritairement à travers le suivi psycho-social (58,3%).

**Aptitudes et pratiques en matière de réintégration sociale des FVFO:**contrairement aux autres activités économiques, le commerce génère rapidement et facilement de l'argent. Ce qui explique en partie le fait que le fonds de commerce soit l'aide la plus sollicitée (62,5%) parmi les femmes opérées. En effet, les FVFO ont besoin d'argent pour une utilisation quotidienne (logement, nutrition, vêtement) mais aussi pour payer d'autres soins de santé liés aux problèmes sous-jacents àla FO, comme les infections uro-génitales causées par la fuite incontrôlée et permanente des urines et des selles [32, 33]. Ce constat est assez différent lorsqu'on considère tous les avis des FVFO (opérées ou non) en matière de pratique de la réintégration sociale. A ce niveau la majorité des femmes optent pour une activité moins contraignante: l’élevage (64,3%). En effet, la FO est une pathologie invalidante non seulement à cause de la douleur chronique qui empêche la femme d'exercer une activité nécessitant beaucoup d'effort physique, mais aussi à cause des mauvaises odeurs permanentes que la femme dégage et qui restreignent fortement sa fréquentation [31–33]. Ainsi, toute activité imposant beaucoup d'effort physique (agriculture) ou impliquant directement des relations interpersonnelles (commerce) est inappropriée et surtout inadaptée au contexte des femmes porteuses de FO. Au-delà des fausses perceptions surla FO qui est considérée comme une punition infligée aux « femmes légères » (infidèles), le rejet dontsont victime les FVFO a été rapporté comme laconséquence des écoulements fréquents et odeurs d'urine, mais aussi comme la conséquence de l'incapacité d'entretenir des relations sexuelles avec leurs conjoints [29, 31, 33, 40]. Ainsi en dépit du fait que 50% et 75% des femmes pensent respectivement que leurs communautés et leurs maris respectifs accepteront les aides, la réussite de la réintégration sociale des FVFO suppose une prise en charge médicale adéquate pour une réparation complète afin que la femme retrouve son estime. Le cas échéant,les femmes serontacceptées par leurs proches une fois retournées dans leurs villages, ce qui sans doute contribuera à les aider à mieux se sentir dans leurs foyers respectifs. Maisdans le cas contraire, la femme sera définitivement désappropriée de son rôle sociale [31, 35, 40]. Ce qui explique en partie le fait que 28% des FVFO déclarent que l'aide reçu ne leur fera pas oublier leur situation de porteuse de fistule. On savait déjà que l'organisation socioculturelle des communautés était l'un des facteurs de risque de la FO [41]. Selon les personnes de l'entourage des FVFO interrogées, c'est aussi une barrière de la réintégration sociale (68,2%). Au niveau des structures de prise en charge des FVFO, il s'agit principalement de la mauvaise gestion de l'aide (41,7%) et de la faible adhérence des femmes au programme de réintégration (25%). C'est pourquoi la méthodologie participative avec implication des autorités traditionnelles et des conjoints est suggérée pour la bonne mise en œuvre des activités de réintégration sociale des FVFO.

## Conclusion

La présente recherche ressort les informations collectées au cours de 92 entretiens auprès de trois groupes d'informateurs: les femmes victimes de fistules obstétricales, leur entourage et les agents de santé. Cette étude fournit les connaissances de base pour la mise en place d'un programme multidisciplinaire de prise en charge de la FO. Egalement à long terme, elle vise à contribuer à l’éradication de la fistule obstétricale dans les zones endémiques. En effet, cette étude s'inscrit en continuité des activités de réparation chirurgicales des FO effectuées en milieu hospitalier. Pour ce faire, les femmes atteintes de fistules qui ont pu être soignées et réhabilitées devront servir d'exemple pour un effet d'entraînement dans leurs communautés d'origine. Une sensibilisation de proximité ciblant les autorités traditionnelles, la communauté en général et en particulier les femmes en âge de procréer contribuera non seulement à l'augmentation du nombre de fréquentation des formations sanitaires, mais aussi à l'adhésion aux programmes de prise en charge et au recrutement des nouveaux cas. La sensibilisation permettra aussi avant tout de délivrer un message de prévention sur les facteurs de risque de survenue de la fistule obstétricale. La prise en charge communautaire de la FO peut être améliorée à travers deux approches: un appui matériel, financier et un suivi psychosocial. Dans le premier cas il s'agira de mettre en place des organisations à base communautaires chargées de suivre les FVFO, leur apporter un soutien matériel (semences et outils agricoles, matériel génétique), promouvoir les programmes d'aides financières en vue de leur permettre d'entreprendre des activités génératrices de revenus et de créer des structures chargées de centraliser les productions pour leur commercialisation. Dans le second cas, il sera question de mettre en place un système de visites à domiciles dans le but d'apporter du réconfort et aider la femme à accepter son statut de porteuse de fistule. Ces réponses locales viennent compléter celles proposées par le gouvernement et ses partenaires pour la prise en charge de la FO, qui aujourd'hui ne semblent plus suffisantes pour soulager définitivement les FVFO. Toutefois, ces approches doivent tenir compte des difficultés auxquels font face les FVFO et des avis de leurs conjoints et entourage.
